# Development of Cost-Effective Sn-Free Al-Bi-Fe Alloys for Efficient Onboard Hydrogen Production through Al–Water Reaction

**DOI:** 10.3390/ma17204973

**Published:** 2024-10-11

**Authors:** Rui Deng, Mingshuai Wang, Hao Zhang, Ruijun Yao, Kai Zhen, Yifei Liu, Xingjun Liu, Cuiping Wang

**Affiliations:** 1 School of Materials Science and Engineering, Harbin Institute of Technology, Shenzhen 518055, China; d434314909@gmail.com (R.D.); 23s136528@stu.hit.edu.cn (H.Z.); cumtyrj1996@126.com (R.Y.); zhkaihit@163.com (K.Z.); l1242070761@163.com (Y.L.); 2 Shenzhen R&D Center for Al-Based Hydrogen Hydrolysis Materials, Shenzhen 518055, China; 3 College of Materials Science and Engineering, Xiamen University, Xiamen 361005, China; 20720191150071@stu.xmu.edu.cn

**Keywords:** aluminum alloy, gas atomization, hydrolysis, hydrogen production, microstructure characterization, activation energy

## Abstract

Leveraging the liquid-phase immiscibility effect and phase diagram calculations, a sequence of alloy powders with varying Fe content was designed and fabricated utilizing the gas atomization method. Microstructural characterizations, employing SEM, EDS, and XRD analyses, revealed the successful formation of an incomplete shell on the surfaces of Al-Bi-Fe powders, obviating the need for Sn doping. This study systematically investigated the microstructure, hydrolysis performance, and hydrolysis process of these alloys in deionized water. Notably, Al-10Bi-7Fe exhibited the highest hydrogen production, reaching 961.0 NmL/g, while Al-10Bi-10Fe demonstrated the peak conversion rate at 92.99%. The hydrolysis activation energy of each Al-Bi-Fe alloy powder was calculated using the Arrhenius equation, indicating that a reduction in activation energy was achieved through Fe doping.

## 1. Introduction

The hydrogen–oxygen fuel cell is increasingly important in various power generators and electricity-consuming devices, including dismounted soldier power supplies [1], automobiles [2], and autonomous undersea vehicles [3]. It is valued for its zero-carbon emissions, quick refueling ability, low signal characteristics, and high energy conservation efficiency. However, the traditional hydrogen refueling routine relies on infrastructure construction, which has become the main obstacle to further fuel cell applications. Therefore, the concept of “hydrogen production onboard” has gained extensive attention due to the simplification of hydrogen usage procedures. Several onboard hydrogen production methods such as metal hydrolysis [4], metal hydride hydrolysis [5] or pyrolysis [6], methanol steam reforming [7], and sodium borohydride hydrolysis [8,9] have been proposed to achieve this concept. Among these methods, Al hydrolysis has been considered the most practical way to generate hydrogen on demand due to its affordable hydrogen production cost, high hydrogen storage density, and simplicity in usage.

Although the Al–water reaction is thermodynamically favorable, producing hydrogen at a sufficient rate for practical applications remains a challenge. The formation of a dense oxide passivation layer on the surface of Al prevents contact between Al and water and severely reduces the reaction’s activity [10,11]. One effective way to improve the hydrolysis activity of Al is by adding alkaline [12], which dissolves the oxide passivation layer and Al-containing reaction products. However, this method poses a risk of equipment corrosion and environmental pollution. To continuously produce hydrogen through the Al hydrolysis reaction in a neutral solution, various additives such as metals [13,14], salts [15], oxides [16], hydride [17], and graphite [18] have been introduced into Al via alloying, high-energy ball milling, or atomization methods, and these techniques show great potential in promoting the Al–water reaction.

Woodall [19,20] discovered that certain Al alloys with liquid phases, such as Al-Ga [21] and Al–Ga–In–Sn [22], can induce the Al–water reaction in a neutral solution at room temperature, resulting in a significant rate of hydrogen production. The liquid regions within the alloys act as channels for Al diffusion, enabling internal Al atoms to breach the passive oxide barrier and react with water [23]. Following these findings, a number of studies have employed Al-Ga-based alloy hydrolysis reactions to generate hydrogen onboard. Wang et al. [24] analyzed the hydrogen production performance of a range of multicomponent low-melting-point Al alloys containing Al, Ga, In, and Sn, and the results indicated that quaternary Al alloys outperformed binary and ternary Al alloys. Jin et al. [25] investigated the effects of additive In and Sn on the hydrolysis performance of an Al–Ga-based alloy. They discovered that In causes more severe cracks to form on the surface of the alloy, while Sn tends to collect more Ga to create a low-melting-point phase at the grain boundary. This implies that In yields better hydrogen production, while Sn yields a higher rate of hydrogen production. However, the low-melting-point alloying elements Ga and In are very expensive, making them unaffordable for practical applications.

Previous studies conducted by our research group have demonstrated a novel method for enhancing the hydrolysis activity of Al-Bi-Sn-based alloy powder, excluding Ga and In doping, through atomization [26,27]. Leveraging the liquid-phase immiscibility effect, an incomplete shell composed of (Bi, Sn)-rich phases formed over the powder surface, disrupting the continuous passive oxide layer of Al and causing cracks [28]. As a result, the Al-Bi-Sn-based alloy powder demonstrated excellent hydrogen production efficiency. Although the cost of producing hydrogen through Al-Bi-Sn-based alloy powder is significantly lower than that of an Al-Ga-based alloy, the use of Sn remains cost-prohibitive for practical applications. To address this issue, our research group [29,30] explored the use of low-cost alloy elements, such as Cu and Fe, to reduce Sn content. A series of quaternary Al-Bi-Sn-X (Cu and Fe) alloy powders, with the X element added in varying proportions ranging from 0 to 3 wt.%, were fabricated and tested. The results revealed that while Cu suppresses hydrolysis in Al-Bi-Sn-based powders, Fe enhances it, indicating that replacing Sn with a cheaper element can be an efficient way to lower the cost of hydrogen production.

Despite reducing Sn content in previous works, it remains a primary alloying element (up to 7 wt.%) to ensure adequate hydrolysis properties, limiting large-scale applicability. To overcome this challenge, this study employed phase diagram calculations to design a series of Sn-free, cost-effective Al-Bi-Fe alloy powders. These powders were produced via atomization and thoroughly characterized for their microstructure, hydrolysis performance, and reaction kinetics. The findings demonstrate that appropriate Fe additions significantly enhance hydrogen generation in Sn-free Al-Bi-based alloys.

## 2. Materials and Methods

### 2.1. Material Preparation

In this study, Al-10Bi-xFe (x = 3, 7, 10) wt.% alloy powders were prepared using the gas atomization method. The raw materials, comprising high-purity (99.9%) bulk metals of Al, Bi, and Fe, underwent controlled melting within a high-frequency induction melting furnace under argon protection. Subsequently, the molten alloy was atomized through a high-pressure argon flow ranging from 5 to 8 MPa. The atomized powders were efficiently collected, and any abnormally coarse particles were filtered out with sieves in the argon environment. The resulting powder was stored in labeled glass bottles for subsequent experiments.

### 2.2. Hydrolysis Test

**Figure 1:**
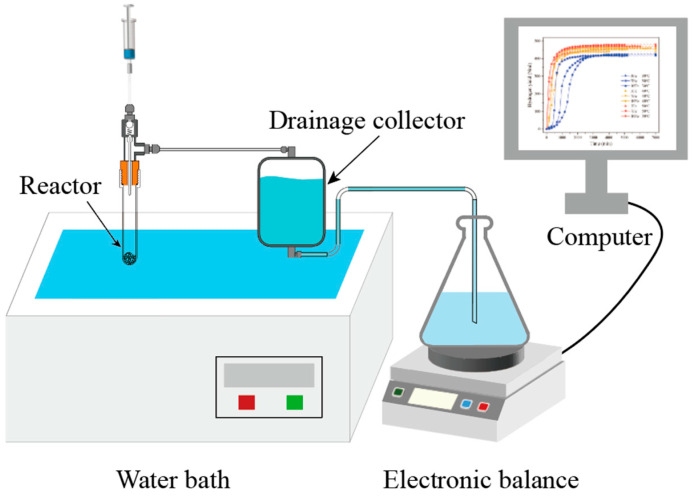
The schematic experimental setup for the hydrolysis test.

The hydrogen volume was measured using the drainage method, and the experimental setup for the hydrolysis test is illustrated in Figure 1. Hydrolysis tests for Al-Bi-Fe variations were carried out at different reaction temperatures ranging from 30 to 50 °C, employing deionized water. Both the reactor and drainage collector were immersed in a water bath at the reaction temperature. Throughout the test, 10 mL of deionized water at the reaction temperature was injected into a meticulously designed reactor containing 0.5 g of Al-Bi-Fe alloy powders. A computer connected to the electronic balance recorded the weight change. To ensure comparability of the recorded data between tests at different reaction temperatures, the volume of hydrogen yield was normalized under standard conditions (0 °C, 1 atm) with the unit of NmL.

### 2.3. Characterization

As-atomized powders were mounted, ground, and polished for preparing specimens of the cross-section of the powders. The microstructure was characterized by using a Quanta 650 FEG scanning electron microscope from Thermo Fisher Scientific, based in Waltham, MA, USA. This SEM, operated at 15 kV, was also equipped with energy dispersive spectroscopy (EDS) for a semi-qualitative elemental analysis. A Rigaku Rapid IIR X-ray diffractometer (XRD) from Rigaku Corporation, located in Tokyo, Japan, was utilized with Cu-K a radiation at a step rate of 2° per minute to analyze the crystal structure of the as-atomized powders.

## 3. Results and Discussion

### 3.1. Design of Powder Composition

**Figure 2:**
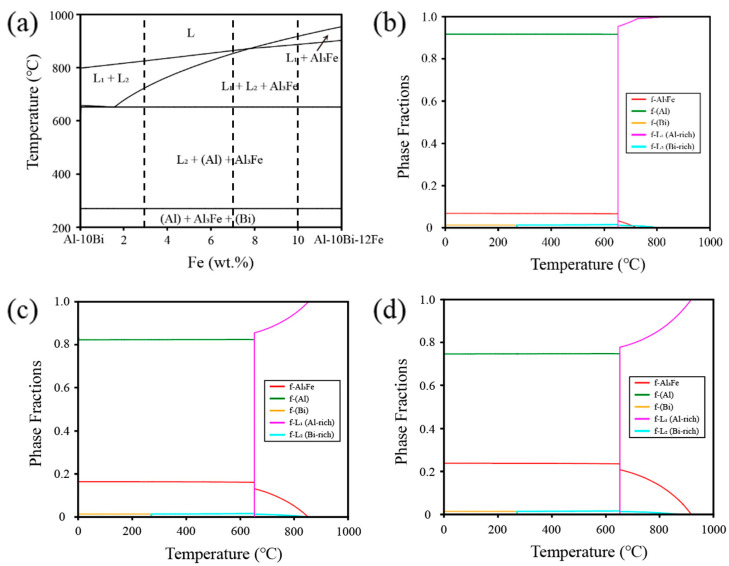
(**a**) Calculated vertical phase diagrams of Al-10Bi-Fe (wt.%); (**b**–**d**) calculated phase fractions diagrams of Al-10Bi-(3, 7, 10) Fe (wt.%).

The phase diagram calculations of the ternary Al-Bi-Fe alloy were conducted using the Pandat software (version 2019) based on the Al alloy database, and the results are presented in Figure 2. Notably, Figure 2a shows a distinct liquid-phase miscibility region labeled as (L1 + L2) or (L1 + L2 + Al_3_Fe). This suggests that the alloy has the potential to form an incomplete shell without the presence of Sn, leading to high activity in the Al–water reaction. Subsequently, a series of Al-Bi-Fe alloys with Fe additions varying from 3 to 10 wt.% was designed, and their calculated phase fraction diagrams are depicted in Figure 2b–d. As shown in the figures, the types of phases in the alloys remain relatively unchanged despite variations in Fe content. Detailed phase equilibrium calculations for Al-10Bi-3Fe, Al-10Bi-7Fe, and Al-10Bi-10Fe (wt.%) at 700 °C, within the range of the liquid-phase miscibility gap, were conducted, and the results are provided in Table 1. The calculations suggest that the phase fraction of L2(Bi-rich) and Al_3_Fe increases with Fe content, while L1(Al-rich) decreases. To explore the influence of phase fraction variation on hydrolysis performance, these alloy powders were fabricated and tested.

### 3.2. Microstructure Characterization of As-Atomized Powders

**Figure 3:**
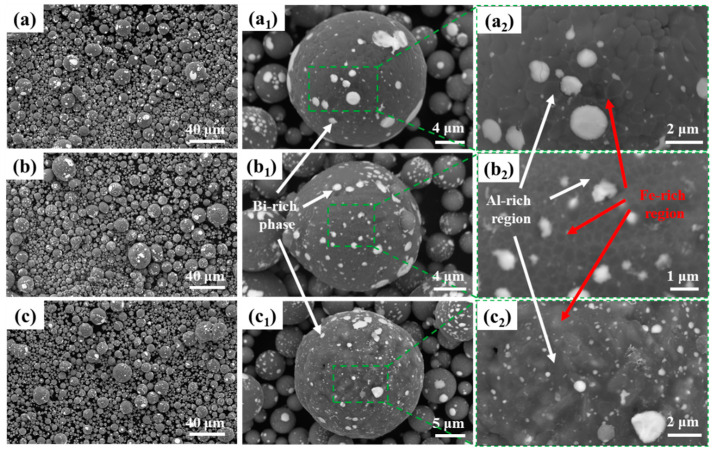
SEM images at various magnifications of as-atomized Al-Bi-Fe alloy powders: (**a**–**a_2_**) Al-10Bi-3Fe (wt.%); (**b**–**b_2_**) Al-10Bi-7Fe (wt.%); and (**c**–**c_2_**) Al-10Bi-10Fe (wt.%).

The as-atomized powder particles and surface morphology of Al-10Bi-3Fe, Al-10Bi-7Fe, and Al-10Bi-10Fe (wt.%) were characterized using SEM, as illustrated in Figure 3. The particles exhibit good sphericity, with sizes primarily ranging from 1 to 50 μm. Due to the liquid-phase miscibility effect and the significant phase fraction difference between the Al-rich (L1) and Bi-rich (L2) phases, the white Bi-rich phase is dispersed across the powder surface, as shown in Figure 3(a_1_–c_1_). The results suggest that an incomplete shell of the Bi-rich phase forms on the surface of these powders, potentially disrupting the continuous oxide layer and promoting favorable hydrolysis activity [28].

Figure 3(a_2_–c_2_) presents magnified SEM images of the regions outlined by green dashed lines, revealing two distinct matrix regions with varying contrasts. The whiter matrix region increases with Fe content, indicating a tendency for Fe segregation. While few Fe-rich regions are observed at the grain boundaries of Al-10Bi-3Fe, a notable number are present in Al-10Bi-7Fe. As the Fe content increases to 10 wt.%, the Fe-rich regions expand, indicating a more inhomogeneous Fe distribution with higher Fe content.

**Figure 4:**
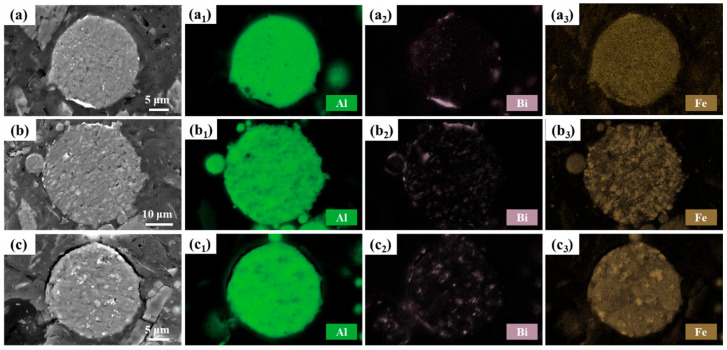
EDS analysis of the cross-section of the Al-Bi-Fe alloy powders: (**a**) Al-10Bi-3Fe, with element distributions: (**a_1_**) Al; (**a_2_**) Bi; (**a_3_**) Fe; (**b**) Al-10Bi-7Fe (wt.%), with element distributions: (**b_1_**) Al; (**b_2_**) Bi; (**b_3_**) Fe; (**c**) Al-10Bi-10Fe (wt.%), with element distributions: (**c_1_**) Al; (**c_2_**) Bi; (**c_3_**) Fe.

Further characterization was conducted to unveil the phase distribution in powder variations. Figure 4 illustrates the cross-sectional morphology of Al-10Bi-3Fe, Al-10Bi-7Fe, and Al-10Bi-10Fe (wt.%), showcasing the element distribution in these plates. In Figure 4(a_2_–c_2_), it is observed that the Bi-rich phase is primarily distributed on the surface of the powders, forming an incomplete shell that encloses the Al-rich phase within the powders. The remaining Bi-rich phase is dispersed throughout the matrix. Additionally, the amount of Bi-rich phase accumulated on the surface decreases with increasing Fe content, indicating that the solidification of Fe atoms impedes the separation of L1 (Al-rich) and L2 (Bi-rich), resulting in a relatively homogeneous distribution of the Bi-rich phase. 

Fe is homogeneously distributed throughout the cross-section, without obvious preferential segregation in the Al-10Bi-3Fe powder (Figure 4(a_3_)). However, Fe demonstrates segregation tendencies in high Fe-containing powders, forming several Fe-rich regions in the matrix, consistent with observations in Figure 3(a_2_–c_2_). Notably, the formation of the secondary Al_3_Fe phase appears minimal in the powder variations, contradicting the calculated phase diagrams. This discrepancy can be attributed to the high solubility of Fe in Al and the rapid cooling rates achieved during atomization, which significantly inhibit the formation of the Al_3_Fe phase.

**Figure 5:**
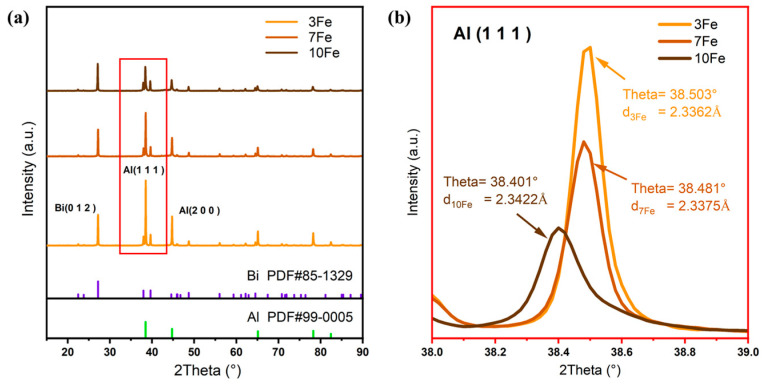
XRD patterns of the as-atomized Al-Bi-Fe alloy powders (**a**) separated XRD patterns; (**b**) stacked patterns focusing on the Al (111) peak.

Figure 5 depicts the XRD patterns of the as-atomized Al-Bi-Fe alloy powders. In addition to the discernible Al and Bi diffraction peaks, Al_3_Fe peaks are scarcely detected, indicating significant Fe solid solution and the absence of the Al_3_Fe phase. This observation is consistent with the EDS analysis, which revealed minimal secondary phases other than the Bi-rich phase within the powder cross-sections. Thus, the regions where Fe segregates are coherent with the Al matrix, indicating two distinct Fe solidification regions (Al-rich and Fe-rich) within the matrix, as shown in Figure 4(b_3_,c_3_).

As the Fe content increases, the Bi diffraction peaks remain unchanged, while the Al diffraction peaks decrease, suggesting that Fe doping mitigates the surface segregation tendency of Bi, aligning with the EDS observations (Figure 4(a_2_–c_2_)). Additionally, the lattice parameters for Al-10Bi-3Fe, Al-10Bi-7Fe, and Al-10Bi-10Fe were calculated from the XRD patterns for the Al (111) peak using Jade software, yielding values of 2.3362 Å, 2.3375 Å, and 2.3422 Å, respectively, as shown in Figure 5b. These results further demonstrate that increasing the Fe content leads to more pronounced Fe solid solution, as Fe atoms have larger atomic radii than Al atoms.

### 3.3. Hydrolysis Performance

**Figure 6:**
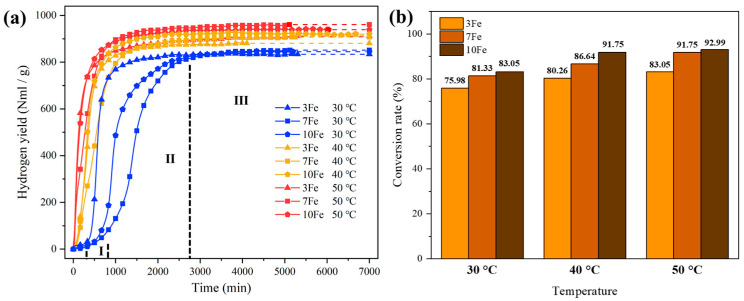
Charts of the hydrolysis of Al-Bi-Fe variations using deionized water at different reaction temperatures ranging from 30 to 50 °C: (**a**) the hydrogen yield vs. time curve; (**b**) the conversion rate.

Figure 6 illustrates the hydrogen yield versus time curves and conversion rates for the hydrolysis of Al-Bi-Fe variations in deionized water at reaction temperatures ranging from 30 to 50 °C. The hydrolysis process of as-atomized Al-based alloy powders generally consists of three distinct periods, the incubation period (Period I), the rapid reaction period (Period II), and the faded reaction period (Period III), as noted in previous studies [27,29,30]. These three stages are evident for Al-10Bi-7Fe in Figure 6a. Initially, during the incubation period, the hydrogen generation rate is negligible. This is followed by an acceleration in hydrogen generation during the rapid reaction period, which leads to substantial hydrogen accumulation. Finally, the hydrogen generation rate slows down and approaches zero in the faded reaction period.

A noteworthy observation is the non-monotonic hydrolysis behavior of Al-Bi-Fe variations with increasing Fe content, particularly with Al-10Bi-7Fe, which exhibits the longest incubation period and the slowest hydrogen generation rate during the rapid reaction period. This can be attributed to structural changes in the Al-Bi-Fe alloy powders. As discussed above, the increased Fe content enhances Fe solidification and leads to a more homogenous distribution of the Bi-rich phase. The former strengthens the matrix, while the latter reduces the effectiveness of the Bi-rich phase in disrupting the oxide layer, making the alloy powder more resistant to rupture. This extends the incubation period and reduces the hydrogen generation rate during the rapid reaction period.

Conversely, a higher Fe content increases the number of Fe-rich regions, forming micro-galvanic couples with Al-rich regions, which accelerate the hydrolysis process, shortening the incubation period and increasing the hydrogen generation rate during the rapid reaction period. This interplay results in the observed non-linear relationship between Fe content and hydrolysis behavior in Al-Bi-Fe variations.

In the Al-10Bi-7Fe alloy, Fe solidification and the second phase strengthening effect are more pronounced compared to the Al-10Bi-3Fe alloy, while fewer micro-galvanic couples form compared to the Al-10Bi-10Fe alloy. These factors collectively result in the longest incubation period and the slowest hydrogen generation rate during hydrolysis.

**Figure 7:**
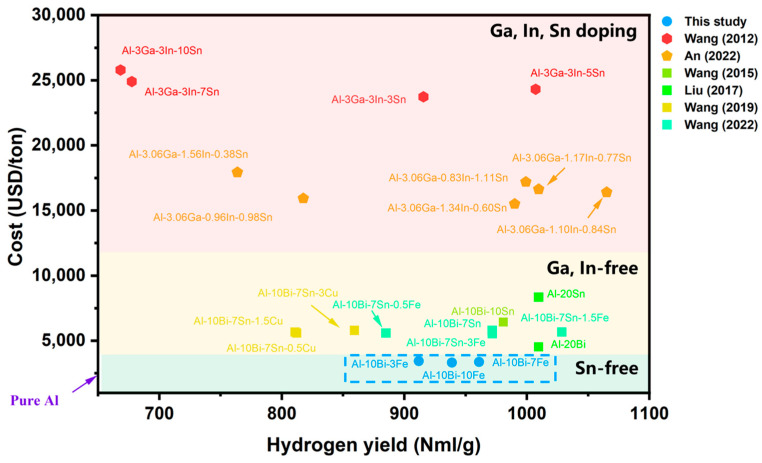
Cost and hydrolysis performance for various active Al alloys: (1) red area indicates Ga, In, Sn doping alloys; (2) yellow area indicates Ga, In-free alloys; and (3) green area indicates Sn-free alloys. The data are drawn from references [23,24,26,27,29,30].

As reaction temperature increases, the hydrolysis kinetics are enhanced across all Al-Bi-Fe variations, improving overall performance. At 50 °C, Al-10Bi-7Fe produces the highest hydrogen yield (961.0 NmL/g), while Al-10Bi-3Fe and Al-10Bi-10Fe generate 911.8 NmL/g and 938.8 NmL/g, respectively. Figure 6b shows the conversion rates for each variation, indicating that both higher reaction temperatures and the increased Fe content boost the conversion rates. Al-10Bi-10Fe, with the highest Fe content, achieves a remarkable conversion rate of 92.99%, surpassing Al-10Bi-3Fe and Al-10Bi-7Fe, which reach 83.05% and 91.75%, respectively, at 50 °C. Despite Al-10Bi-10Fe’s higher conversion rate, its hydrogen yield is lower than that of Al-10Bi-7Fe due to a smaller amount of hydrolyzable Al. Figure 7 presents a comparison of the cost and hydrolysis performance of various active Al alloys at temperatures near 50 °C, with detailed data provided in Table S1.

The extent of Fe doping plays a critical role in enhancing the conversion rate, as it alters the Bi-phase distribution. With higher Fe doping, more Bi phase is distributed within the matrix rather than on the surface, creating favorable conditions for powder rupture, as highlighted in previous studies [28,31]. This leads to more thorough hydrolysis, improving the conversion rate for Al-Bi-Fe variations with elevated Fe content.

### 3.4. Chemical Kinetics of Hydrolysis Reaction

**Figure 8:**
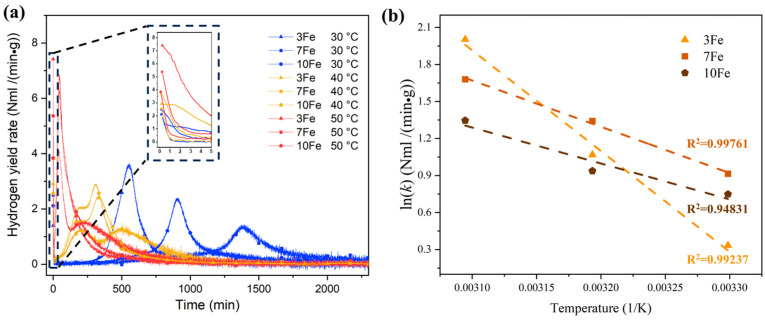
Plots of the hydrolysis of the Al-Bi-Fe variations using deionized water at different reaction temperatures ranging from 30 to 50 °C (**a**) hydrogen yield rates vs. time curves, (**b**) the Arrhenius plots.

Figure 8a illustrates the hydrogen yield rate (*k*) vs. time curves of the hydrolysis reaction of Al-Bi-Fe variations. These curves are derived by taking the derivative of the curves presented in Figure 6a and applying smoothing using the Savitzky–Golay method with a window size of 12 (the interval between data points is 5 s) and a polynomial order of 1. The initial hydrogen yield rates of Al-Bi-Fe variations are observed even at a reaction temperature of 30 °C due to the immediate reaction of the as-atomized powders with sufficiently small sizes when exposed to deionized water [32]. This occurrence takes place despite the existence of the incubation period. As shown in the inset of Figure 8a, the initial hydrogen yield rates of Al-Bi-Fe variations are strongly relevant to the reaction temperature. The Arrhenius equation is used to model the initial hydrogen yield rates of THE hydrolysis reaction and for calculating the activation energy of Al-Bi-Fe variations; the formula is expressed as follows [33,34]:

(1) $$\ln\left(k\right)=\frac{-E_{a}}{R}\left(\frac{1}{T}\right)+ln(A)$$

Here, *k* is the hydrogen yield rate of Al-Bi-Fe variations (NmL/(min·g)), *Ea* is the activation energy for the hydrolysis reaction (J/mol), *R* is the universal gas constant (8.314 J/(mol·K)), *T* is the absolute temperature (K), and *A* is the Arrhenius factor.

Figure 8b depicts the Arrhenius plots of Al-Bi-Fe variations in deionized water. The plots demonstrate a significant linear correlation between ln(k) and 1/T across all Al-Bi-Fe variations, and the calculated activation energy values are listed in Table 2. As indicated in the table, the activation energy of the as-atomized Al-10Bi-3Fe, Al-10Bi-7Fe, and Al-10Bi-10Fe powders is 67.888, 31.178, and 24.224 kJ/mol, respectively. This suggests a reduction in activation energy achieved by doping Fe in the Al-Bi-based alloy.

### 3.5. Hydrolysis Process

**Figure 9:**
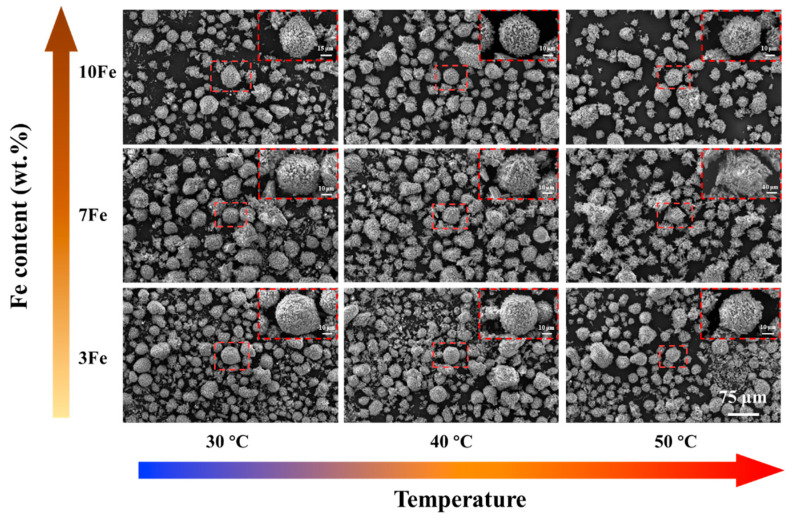
SEM images of the hydrolysis products of Al-Bi-Fe variations using deionized water at different reaction temperatures ranging from 30 to 50 °C.

SEM images of the hydrolysis products from Al-Bi-Fe variations exposed to deionized water at reaction temperatures ranging from 30 to 50 °C are shown in Figure 9. As observed in the figure, powders with minimal sizes nearly undergo complete dissolution, whereas larger particles undergo extensive hydrolysis, as depicted in the inset with the red dashed line. This observation underscores the remarkable hydrolysis efficacy of Al-Bi-Fe alloy powders at moderate temperatures, revealing the promising potential for hydrogen production from Sn-free Al-Bi-based alloys.

**Figure 10:**
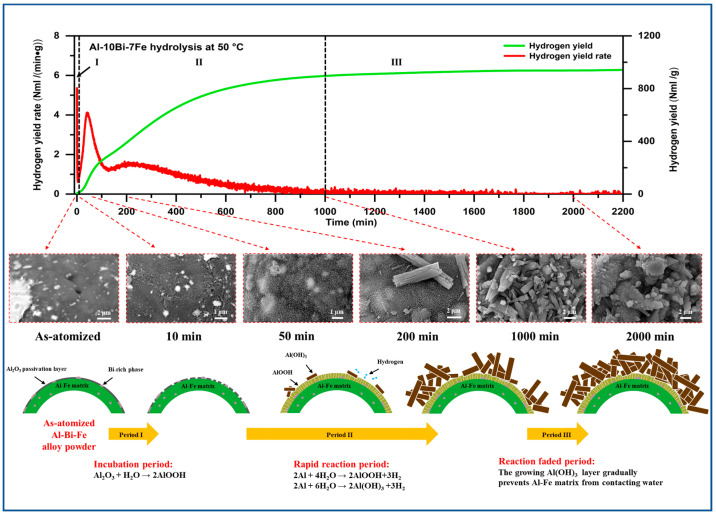
The morphological changes on the surface of Al-Bi-Fe alloy powders at different stages of the hydrolysis process, along with a schematic representation of the reaction mechanism.

To investigate the hydrolysis behavior of Al-Bi-Fe alloy powders during each reaction period, a series of Al-10Bi-7Fe powders were subjected to hydrolysis in deionized water for varying reaction times, and the resulting hydrolysis products were characterized using SEM. Figure 10 shows the morphological changes on the surface of these products at different stages of the hydrolysis process. 

During the incubation period, the Al_2_O_3_ passive layer is hydrated without hydrogen generation, resulting in pitting on the powder surface [35], as shown in the SEM image for a reaction time of 10 min, according to Equation (2):

Al_2_O_3_ + H_2_O → 2AlOOH (2)

Once the Al_2_O_3_ passive layer breaks down, water begins to react with the Al-Fe matrix, marking the onset of the rapid reaction period. During this phase, water reacts with the Al-Fe matrix according to Equation (3) until the powder surface is covered by stacked AlOOH platelets [36,37], as seen in the SEM image for a reaction time of 50 min. The hydrolysis then follows Equation (4), resulting in the formation of columnar Al(OH)_3_ [38], as shown in the SEM image for a reaction time of 200 min.

2Al + 4H_2_O → 2AlOOH + 3H_2_ (3)

2Al + 6H_2_O → 2Al(OH)_3_ + 3H_2_ (4)

Both hydrolysis processes generate a significant amount of hydrogen. The columnar Al(OH)_3_ layer grows rapidly during the rapid reaction period and eventually almost completely inhibits the contact between the Al-Fe matrix and water, as seen in the SEM image for a reaction time of 1000 min. Consequently, the hydrolysis process enters the faded reaction period, during which the hydrogen yield rate gradually declines to zero as the coarsening columnar Al(OH)_3_ on the powder surface prevents further contact between the Al-Fe matrix and water, as seen in the SEM image for a reaction time of 2000 min. The complete hydrolysis process of the Al-Bi-Fe alloy powders is detailed in Figure S1 and schematically represented in Figure 10.

## 4. Conclusions

In summary, a series of Al-Bi-Fe alloy powders was meticulously designed and prepared using the gas atomization method. Microstructural characterizations unveiled the successful fabrication of an incomplete shell comprised of the Bi-rich phase on the powder surfaces, achieved without Sn doping. Increased Fe content enhances the solidification strengthening effect, alters the distribution of the Bi-rich phase, and promotes the formation of micro-galvanic couples. The intricate interplay of these factors results in significant hydration during hydrolysis and contributes to the excellent hydrolysis performance of these alloy powders. Specifically, Al-10Bi-7Fe exhibited the highest hydrogen yield, reaching 961.0 NmL/g, while Al-10Bi-10Fe demonstrated the peak conversion rate at 92.99%. The reduction in activation energy achieved through Fe doping, along with the observed microstructural changes, substantiates the valuable potential of these alloys for efficient and cost-effective hydrogen generation.

## Figures and Tables

**Table 1:** Calculated phase fraction of Al-Bi-Fe variations at 700 °C.

Alloys (wt.%)	L1(Al-Rich)	L2(Bi-Rich)	(Al)	(Bi)	Al_3_Fe
Al-10Bi-3Fe	0.982	0.006	0	0	0.012
Al-10Bi-7Fe	0.841	0.015	0	0	0.144
Al-10Bi-10Fe	0.791	0.017	0	0	0.192

**Table 2:** Hydrogen yield rate and activation energy of the Al-Bi-Fe variations.

Alloys (wt.%)	Initial Hydrogen Yield Rate at 30 °C (NmL H_2_/(min·g))	Initial Hydrogen Yield Rate at 40 °C (NmL H_2_/(min·g))	Initial Hydrogen Yield Rate at 50 °C (NmL H_2_/(min·g))	Activation Energy (kJ/mol)
Al-10Bi-3Fe	1.396	2.910	7.412	67.888
Al-10Bi-7Fe	2.496	3.826	5.363	31.178
Al-10Bi-10Fe	2.114	2.551	3.842	24.224

## Data Availability

The original contributions presented in this study are included in the article. Further inquiries can be directed to the corresponding author.

## Supplementary Materials

The following supporting information can be downloaded at: https://www.mdpi.com/article/10.3390/ma17204973/s1, Figure S1: The morphological changes on the surface of Al-Bi-Fe alloy powders at different reaction times of the hydrolysis process; Table S1: Cost and hydrolysis performance data for various active Al alloys.
