# Supplementary material for: Development of Cost-Effective Sn-Free Al-Bi-Fe Alloys for Efficient Onboard Hydrogen Production through Al–Water Reaction
Source: Materials (Basel). 2024 Oct 11;17(20):4973. doi: 10.3390/ma17204973 (PMC11509418; doi:10.3390/ma17204973)
Supplement: Supplementary file 1 [file materials-17-04973-s001.zip › materials-3247960-supplementary.pdf]

Supplemental Materials for:

Development of cost-effective Sn-free Al-Bi-Fe alloys for efficient onboard hydrogen production through Al-Water reaction

Table S1 Cost and hydrolysis performance data for various active Al alloys

Alloys (wt.%)	Fabrication method	Cost ($/ton)	Reaction temperature (°C)	Hydrogen yield (Nml/g)	Source
Al-10Bi-3Fe	Atomization	3435.01	50	911.78	This study
Al-10Bi-7Fe	Atomization	3364.60	50	961.00	This study
Al-10Bi-10Fe	Atomization	3311.79	50	938.77	This study
Al-3Ga-3In-3Sn	Ball milling	23726.20	RT	915.54	[23]
Al-3Ga-3In-5Sn	Ball milling	24315.68	RT	1007.09	[23]
Al-3Ga-3In-7Sn	Ball milling	24905.16	RT	677.50	[23]
Al-3Ga-3In-10Sn	Ball milling	25789.38	RT	668.34	[22]
Al-3.06Ga-1.56In-0.38Sn	Cast	17929.25	50	763.64	[22]
Al-3.06Ga-1.34In-0.60Sn	Cast	17197.32	50	999.11	[22]
Al-3.06Ga-1.17In-0.77Sn	Cast	16631.75	60	1009.65	[22]
Al-3.06Ga-1.10In-0.84Sn	Cast	16398.86	70	1065.10	[22]
Al-3.06Ga-0.96In-0.98Sn	Cast	15933.09	50	817.51	[22]
Al-3.06Ga-0.83In-1.11Sn	Cast	15500.59	60	989.99	[22]
Al-10Bi-10Sn	Atomization	6435.23	50	980.77	[25]
Al-20Bi	Atomization	4522.06	50	1009.54	[26]
Al-20Sn	Atomization	8348.41	50	1009.54	[26]
Al-10Bi-7Sn-0.5Cu	Atomization	5591.58	50	812.11	[28]
Al-10Bi-7Sn-1.5Cu	Atomization	5672.72	50	810.75	[28]
Al-10Bi-7Sn-3Cu	Atomization	5794.43	50	859.24	[28]
Al-10Bi-7Sn-0.5Fe	Atomization	5591.58	50	884.92	[29]
Al-10Bi-7Sn-1.5Fe	Atomization	5672.72	50	1028.46	[29]
Al-10Bi-7Sn-3Fe	Atomization	5794.43	50	971.68	[29]
Al-10Bi-7Sn	Atomization	5551.01	50	971.77	[29]

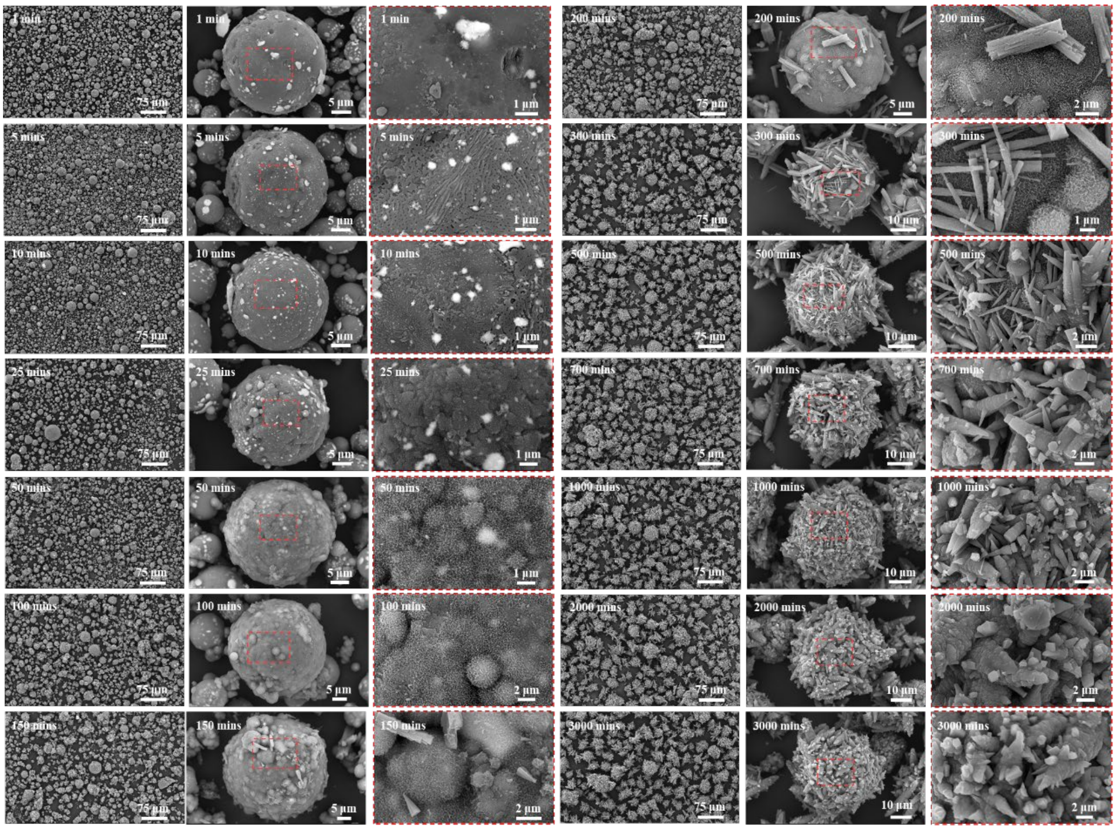

Figure S1 Morphological changes on the surface of Al-Bi-Fe alloy powders at different reaction time of the hydrolysis process
